# Correlates of abortions and condom use among high risk women attending an std clinic in st Petersburg, Russia

**DOI:** 10.1186/1742-4755-8-28

**Published:** 2011-10-12

**Authors:** Nadia Abdala, Weihai Zhan, Alla V Shaboltas, Roman V Skochilov, Andrei P Kozlov, Tatiana V Krasnoselskikh

**Affiliations:** 1 Yale School of Public Health, 60 College Street, New Haven, CT, 06520, USA; 2 The Biomedical Center, 8 Vyborgskaya ul, St. Petersburg, 194044, Russian Federation; 3 Saint Petersburg, State University, 7/9 Universitetskaya nab, St Petersburg, 199034, Russian Federation; 4 Pavlov State Medical University, 6/8 Leo Tolstoy Str., St Petersburg, 197022, Russian Federation

**Keywords:** abortion, condom use, Russia, HIV risk, high risk women, alcohol misuse, AUDIT-C

## Abstract

**Background:**

Many women in Russia rely on abortion as a primary birth control method. Although refusal to use contraceptives, including condoms, may undermine public health efforts to decrease HIV sexual risk behaviors, few studies have investigated the risk factors associated with abortion among women at high risk for HIV. This study sought to identify the correlates of abortions and of lack of condom use among high risk STD clinic patients in St Petersburg Russia.

**Methods:**

Cross-sectional analysis of data collected between 2009 and 2010 from women who had casual or multiple sexual partners in the previous three months was analyzed. Multivariate logistic regression assessed the independent correlates of abortion(s) and no condom use in the prior three months. Independent variables included socio-demographics, at risk drinking per alcohol use disorder identification test (AUDIT-C) criteria, having sex after drinking alcohol, having a sexual partner who injects illicit drugs, and parity.

**Results:**

Of 87 participants, 45% had an abortion in their lifetime and 26% did not use condoms in the prior three months. Abortion was independently associated with low income (OR, 3.33, 95%CI, 1.13-9.78) and at risk drinking (OR, 3.52, 95%CI, 1.24-10.05). Lack of condom use was independently associated with being more likely to have sex after drinking (OR, 3.37, 95%CI, 1.10-10.28) and parity (OR, 3.69, 95%CI, 1.25-10.89).

**Conclusions:**

Programs to increase contraceptive use including condom use among women at high risk for STD/HIV in Russia are needed. Programs to reduce sexual HIV risk and abortion rates must address alcohol misuse and target women with limited income.

## Introduction

Abortions were the primary means of birth control in the Soviet Union [1,2], and abortion rates in Russia are among the highest in the world today. Data from the United Nations Statistics Division from 2003 to 2004, show an abortion rate in Russia of 54% per 1,000 women aged 15 to 44, which is more than twice the rate in the United States (21% per 1,000 women aged 15 to 44) and the highest of the Eastern and Western European countries [3]. Studies in Russia show that abortion prevalence as reported by women can range from 50% to 75% in different cities [4,5]. Although efforts to decrease unwanted pregnancy and abortion rates in the mid-1990s [6-9] have led to a reduction of abortions and better attitudes toward contraceptive methods in Russia [9], recent studies show that some Russian women still prefer not to use contraceptives [4,10] and may rely on abortions as a method of birth control [10,11].

Women who use abortions as a means to prevent births are particularly vulnerable to HIV in Russia as the epidemic spreads from a predominantly male population who inject drugs to their sexual partners [12]. Data from the Russian Ministry of Health indicate that 3% of new HIV infections among women were acquired during unprotected sex with infected men [13]. Moreover, the refusal to use contraceptives including condoms may undermine efforts of programs to reduce sexual risk behaviors among women. For these reasons, investigations of risk factors for abortions and unsafe sex among high risk populations in Russia may provide useful information for interventions to reduce human immunodeficiency virus (HIV) risk and unwanted pregnancies and to improve the reproductive health of women.

Although data consistently show that abortions are common in Russia [10], studies of the factors associated with abortions show contradictory findings. In one Russian study conducted among the general population the women who reported having multiple sexual partners were generally more likely to have abortions [4], whereas in a study among female injection drug users in St Petersburg, the women who had multiple sexual partners were less, not more likely to have abortions [14]. Population surveys suggest that specific risk factors may place women at greater risk for abortions, such as having first intercourse below age 18, having lower number of years of education [4], or having economic constraints [11], but these risk factors for abortion have not been investigated in samples of women at high risk for HIV. Moreover, most studies of abortions in Russia have not investigated whether substance use related factors may play a role in abortions. Data show that the rates of alcohol use among Russian women can be high [15-17]. Alcohol misuse, which has been associated with behaviors that place women at greater risk for sexually transmitted diseases (STDs)/HIV, such as unplanned sexual intercourse, unprotected sex [18,19] and intimate partner violence [20] may also place women at greater risk for unwanted pregnancies. There is evidence that sexual risk behaviors may be associated with substance use characteristics of one's sexual partner [21].

Despite the need for data, few studies of abortions have been conducted among women at high risk for STDs/HIV in Russia. We conducted an initial study to identify the correlates of abortions and lack of condom use among women attending an STD clinic in St Petersburg, Russia, who reported having multiple or casual sex partners in the last three months.

## Methods

### Study participants and data collection

The present study analyzes the baseline assessment data of a randomized controlled trial of a behavior intervention pilot designed to examine whether a brief intervention can reduce HIV-related risk behaviors of participants. This study was approved by the institutional review boards at the Biomedical Center in St. Petersburg, Russia, and at Yale University in the United States. The study was conducted in a public STD clinic in St. Petersburg, Russia which provides services free of charge or for a nominal fee to patients. Patients seeking STD services were screened for participation in the study. An invitation was made to 338 patients who met the study entry criteria of being at least 18 years old and who reported having two or more sexual partners or at least one casual sexual partner in the three months prior to the interview. Potential participants were informed of the purpose of the study and were assured that the study was confidential and voluntary. A total of 31 patients refused to participate; 307 gave signed informed consent and completed the baseline assessment from July 2009 through November 2010. Of the 307 patients, 87 were women, and they were included in the present analysis.

Data collected included questions on demographics, alcohol use, drug use, HIV/AIDS knowledge and attitudes, sexual risk behaviors, and abortion. Nine questions inquired about participants' attitudes towards condom use, including two that were specifically related to alcohol use in sexual contexts.

Demographic variables included age, sex, marital status, education, and monthly income. A short version of the alcohol use disorders identification test (AUDIT-C) was used to measure alcohol misuse. Participants were asked the following three questions: 1) How often have you had an alcoholic drink in the past three months? 2) How many drinks on average do you typically have on a day when you are drinking? 3) How often in the past three months did you have six or more drinks on one occasion? Each option for each question is allotted a score between 0-4; thus the score range of AUDIT-C is 0-12. Women who presented with scores greater than 2 points were considered more likely to be harmed from drinking (at risk drinking) [22,23]. Because the AUDIT-C measure has not been validated for this particular Russian population, we also analyzed women with AUDIT-C scores greater than 3 points (high risk drinking) and scores greater than 4 points (severe risk) for the sake of comparison. Assessment of expectations about alcohol use concerning sexual relations was based on participants' answers (yes or no) to two questions: 1) Alcohol makes sex more enjoyable, and 2) When I am under the influence of alcohol I agree to have sex more easily. Injection drug use was determined according to the question: "Have you ever injected illicit drugs?" Having an injection drug using (IDU) partner was determined according to another question: "Have you ever had a partner whom you think injected drugs?" Participants were asked how many sexual partners they had in the previous three months. Participants were also asked whether they used condoms and whether they consumed alcohol prior to intercourse in the past three months. The options for these two questions were never, sometimes, in half of the cases, in most of the cases, and always. In the present study, we dichotomized these variables as "never use" or "at least some use." Participants were asked whether they had been pregnant, whether they had delivered a child, whether they had an abortion, and the number of abortions they had. Any participant who reported having had one or more abortions was considered as having had an abortion.

### Statistical analysis

Standard descriptive statistics were used to describe the sample. Bivariate and multivariate logistic regression models were used to determine correlates of abortion as well as predictors of never using condoms. Associations at p ≤ 0.20 were entered into the multivariate logistic regression models. Backward stepwise elimination was used to produce adjusted-odds ratios for variables with significance levels ≤ 0.05. Because at risk drinking, high risk drinking and severe risk drinking are components of AUDIT-C scores, those three covariates were entered into separate multivariate models to avoid collinearity. Data were analyzed by using SAS software (version 9.1, SAS Institute Inc., Cary, NC).

## Results

### Characteristics of study participants

The age of the 87 participants ranged from 18 to 50 years, with a median of 26.0 years (Table 1). Nearly one-third of participants were married, half had at least some higher education, and 41% reported a monthly income < 9,000 rubles (approximately 320 USD), the most frequently endorsed income. Of all participants, half were classified as engaging in at risk drinking per AUDIT-C, nearly half stated that they were more likely to agree to have sex after drinking alcohol, and a third reported finding sex more enjoyable after alcohol consumption. The prevalence of women with AUDIT-C scores for high risk drinking and severe risk drinking were 31% (27/87) and 17% (15/87), respectively (not shown in the table). Although 16.1% of participants had ever had an IDU partner, only 1 of the 87 participants had ever injected drugs herself. In the previous three months, nearly a quarter of participants claimed "never" to use condoms, and 78% reported using alcohol prior to sex at least some of the time. About 63% of the participants had been pregnant, 41% had delivered a child (parity) and approximately 45% had had an abortion.

**Table 1:** Characteristics of high risk^a ^female STD clinic patients from St.Petersburg, Russia (N = 87)

Characteristics	
Demographics	
Age (median (IQR^b^); min 18, max 50)	26 (21-35)
Married	(27/87) 31.0%
Completed college or more	(48/87) 55.2%
Monthly income < 9,000 rubles	(36/87) 41.4%
Alcohol use	
At risk drinking^c^	45/87 (51.7%)
More likely to agree to have sex after drinking	41/87 (47.1%)
Finds sex more enjoyable after drinking	25/87 (28.7%)
Injection drug use	
Ever injected drugs	1/87 (1.1%)
Ever had a sex partner who injected drugs	14/87 (16.1%)
Sexual behavior in the previous three months	
Did not use condoms	23/87 (26.4%)
Used alcohol prior to sex at least some of the times	67/87 (77.7%)
Pregnancy and abortion	
Has ever been pregnant	(55/87) 63.2%
Parity	(36/87) 41.1%
Abortions:	
None	(48/87) 55.2%
1	(18/87) 20.7%
2	(7/87) 8%
3 or more	(13/87) 16.1%

^a ^i.e., reported having multiple or casual sexual partners in the last 3 months

^b ^IQR = interquartile range

^c ^i.e., received an "at risk" score in the alcohol use disorder identification test (AUDIT-C)

### Correlates of abortion

In the bivariate analysis, age ≥ 26 years [odds ratio (OR), 4.64, 95% confidence interval (CI), 1.86-11.58, p = 0.001], at risk drinking (uOR, 2.50, 95% CI, 1.05-5.97, p = 0.039), being more likely to agree to have sex after drinking (uOR, 3.57, 95% CI, 1.47-8.68, p = 0.005) and parity (uOR, 4.80, 95% CI, 1.92-12.02, p = 0.001) were significantly associated with abortion (Table 2). In the multivariate analysis, only age (aOR, 8.87, 95% CI, 2.83-27.82, p < 0.001), low income (aOR, 3.33, 95% CI, 1.13-9.78, p = 0.029) and at risk drinking (aOR, 3.52, 95% CI, 1.24-10.05, p = 0.018) remained significantly associated with abortion.

**Table 2:** Correlates of abortion among female STD clinic patients, St.Petersburg, Russia (N = 87)

Characteristics	Unadjusted OR^a^ (95% CI)	p-value	Adjusted OR^a^ (95% CI)	p-value
Age (26 years or more)	4.64 (1.86-11.58)	0.001	8.87 (2.83-27.82)	< 0.0001
Married	1.21 (0.49-3.02)	0.680		
Completed college or more	0.75 (0.32-1.76)	0.510		
Monthly income < 9,000 rubles	2.10 (0.88-5.02)	0.093	3.33 (1.13-9.78)	0.029
At risk drinking ^b^	2.50 (1.05-5.97)	0.039	3.52 (1.24-10.05)	0.018
More likely to have sex after drinking	3.57 (1.47-8.68)	0.005		
Finds sex more enjoyable after drinking	2.37 (0.92-6.14)	0.074		
Ever had a sex partner who injected drugs	0.63 (0.19-2.09)	0.460		
Did not use condoms	1.90 (0.72-4.98)	0.192		
Used alcohol prior to sex at least some of the times	1.70 (0.60-4.79)	0.317		
Parity	4.80 (1.92-12.02)	0.001		

^a ^OR, odds ratio; CI, confidence interval.

^b ^i.e., received an "at risk" score in the alcohol use disorder identification test (AUDIT-C)

In the analyses using AUDIT-C with higher cutoff points (not shown in the table), abortion was significantly associated with high risk drinking (uOR, 2.94, 95% CI, 1.15-7.53, p = 0.025) but was not associated with severe risk drinking (uOR, 2.10, 95% CI, 0.68-6.53, p = 0.200). When placed in the multiple logistic regression model instead of at risk drinking, high risk drinking was independently associated with abortion (aOR, 4.03, 95% CI, 1.31-12.30, p = 0.015) together with age ≥ 26 years (aOR, 8.33, 95% CI, 2.70-25.67, p < 0.001) and low income (aOR, 3.57, 95% CI, 1.20-10.62, p = 0.022) whereas severe risk drinking remained nonsignificant.

### Correlates of not using condoms

In the bivariate analysis, being more likely to agree to sex after drinking (uOR, 4.72, 95% CI, 1.64-13.62, p = 0.004) and parity (uOR, 5.03, 95% CI, 1.79-14.14, 0.002) were significantly associated with not using condoms (Table 3). In the multivariate analysis these two variables remained independently associated with not using condoms with aORs of 3.37 (95% CI, 1.10-10.28, p = 0.033) and 3.69 (95% CI, 1.25-10.89, p = 0.018), respectively.

**Table 3:** Correlates of "not using condoms" among female STD clinic patients, St.Petersburg, Russia (N = 87)

Characteristics	Unadjusted OR^a^ (95% CI)	p-value	Adjusted OR^a^ (95% CI)	p-value
Age (26 years or more)^a^	1.30 (0.50-3.40)	0.592		
Married	0.96 (0.34-2.71)	0.940		
Completed college or more	1.10 (0.41-2.81)	0.880		
Monthly income < 9,000 rubles	1.43 (0.55-3.74)	0.460		
At risk drinking ^b^	1.66 (0.63-4.37)	0.310		
More likely to have sex after drinking	4.72 (1.64-13.62)	0.004	3.37 (1.10-10.28)	0.033
Finds sex more enjoyable after drinking	1.47 (0.53-4.10)	0.456		
Ever had a sex partner who injected drugs	0.72 (0.18-2.86)	0.644		
Used alcohol prior to sex at least some of the times	1.10 (0.35-3.47)	0.868		
Had more than one abortions	2.79 (0.98-7.93)	0.055		
Parity	5.03 (1.79-14.14)	0.002	3.69 (1.25-10.89)	0.018

^a ^OR, odds ratio; CI, confidence interval.

^b ^i.e., received an "at risk" score in the alcohol use disorder identification test (AUDIT-C)

The analyses using AUDIT-C with higher cutoff points showed no significant association between not using condoms and high or severe risk drinking (not shown in the table).

## Discussion

This is the first study to investigate whether there is an association between abortions and substance use related factors among women who have casual or multiple sexual partners and are at high risk for HIV and STDs in Russia.

Abortions were independently associated with at risk drinking and with high risk drinking according to AUDIT-C. Although this study could not investigate the reasons for these associations, a few speculations might explain the results. For example, it is possible that these drinkers are less concerned about avoiding abortions, or perhaps they are considering abortion as a birth control method. The latter hypothesis is plausible since studies have shown that some Russian women, particularly disadvantaged ones may be more likely to consider abortion as a birth control method [10,11] and that problematic drinking may be associated with economic strain among Russian men [24,25]. Abortions could also result from the drinkers' greater engagement in risky sexual behaviors leading to a greater chance of an unplanned pregnancy occurring. This is consistent with findings showing that women who drink may be more likely to engage in unplanned intercourse and to report having unplanned pregnancies [26,27]. Studies in Russia have also shown that alcohol consumption may be associated with having multiple sexual partners [28,29] and that abortions may be more common among women who have multiple sexual partners [4]. Studies that investigate the hypotheses presented above are needed in Russia. The findings suggest that interventions to decrease unwanted pregnancies in this population might benefit from addressing alcohol use.

There was no association found between abortion and severe risk drinking. Although this could be an effect of the small sample size studied, it could also be a reflection of the "drinking paradox" [30], which states that certain types of alcohol related harm may primarily be observed among low or moderate drinkers rather than among hazardous drinkers. This effect could be due to lower sexual activity or a relatively lower level of sexual risk among those who engaged in severe patterns of drinking. Although a preference for distilled spirits and a high social tolerance for heavy drinking [16,29] have been thought to partly account for the high rates of alcohol related harm in Russia [17,31], it is possible that, concerning sexual risk taking, heavier drinkers in Russia may be more tolerant to the sedative or disinhibiting effects of alcohol than more moderate drinkers or drinkers from other cultures [32,33]. These findings emphasize the need for the adaptation and validation of measures of alcohol use that are more specific to Russian populations and for larger studies that investigate alcohol and risky sexual behaviors.

Abortions were independently associated with lower income. Although this study did not investigate contraceptive use among participants, abortions might have resulted from a lack of hormonal contraceptive use by participants with lower incomes. In a previous study of women in St Petersburg, Russia, lower socioeconomic status was associated with a lack of hormonal contraceptive use [34]. Another study in Russia found that women who were economically disadvantaged were more likely to resort to repeated abortions and to report economic reasons for those abortions [11]. This study could not compare the characteristics of women who had one abortion to those of females who had repeated abortions because of the restricted sample size. Future studies with larger sample sizes will needed to investigate the factors that encourage women to opt for an abortion or repeated abortions and that influence the choice of contraceptives in the study population. Economically disadvantaged women may lack access to effective contraceptive methods or may face obstacles to obtaining female reproductive health care services. These results indicate a need for intervention programs to increase contraceptive use among these women.

Reports of not using condoms in the previous three months was independently associated with claims of more easily agreeing to have sex after drinking but was not associated with drinking prior to sex or with alcohol misuse (i.e., at risk, high risk or severe risk drinking). Other studies have found such lack of association between alcohol use and unprotected sex [35,36]. Our results indicate that drinking may lead to unprotected sex only among some drinkers or only in some instances of drinking in sexual contexts. The results confirm the need to address alcohol misuse among participants. Future studies using event level approaches should investigate the drinking patterns and contexts that lead women to forgo condoms [35].

The results showing that having previously delivered a child (parity) was independently associated with not using condoms may reflect, for example, a greater economic constraint among women who have children. A study in Russia showing that the financial costs of contraceptive use, including condoms, during one year might be higher than the cost of an abortion [37] suggest that some women may perceive abortions to be economically more viable than the consistent use of condoms. It is also possible that those who have had children may have been using contraceptives other than condoms (e.g., an IUD) or undergone a surgical sterilization procedure. However, being married or having had an abortion was not independently associated with lack of condom use. Future studies will need to investigate the barriers for condom use among women who have children and are at greater risk for STD/HIV.

The 45% prevalence of abortion among participants in this study is similar to the nearly 50% prevalence found in studies among Russian women from the general population between 1973 and 2003 [4,5] and was somewhat lower than the 67% abortion prevalence found among female drug injectors in 2004 in St Petersburg, Russia [14]. Given that this study only recruited female STD clinic patients who reported having casual or multiple sexual partners, the prevalence of abortions and unprotected sex presented in this study are high and suggest that these women are at greater risk for HIV/STDs. These results confirm that there is a need for abortion studies focused on high risk women.

The main limitation of this study is its small sample size, which restricted the analyses that could be conducted and indicates the need for larger studies to confirm these results. We did not ask participants about contraceptive use patterns. The fact that the study sample was highly selective limits the generalizability of the results. The data was based on self-reports of sensitive behaviors during face-to-face interviews, which may have introduced social desirability and recall bias. To our knowledge, the AUDIT-C cutoff scores have not been validated in Russian populations. If Russian women's tolerance for alcohol is greater compared to women from other countries, the results may have overestimated the pattern of alcohol misuse among women. Conversely, since AUDIT-C was based on the question "How often have you had more than six drinks in a row?," this study may have missed the women with lower tolerance for alcohol who engage in alcohol related sexual risk behaviors after drinking fewer than six drinks in a row. However, the study provides a unique opportunity for identification of reproductive health risks that place women at risk for unwanted pregnancies and STD/HIV.

## Conclusion

Abortion was independently associated with alcohol misuse per AUDIT-C criteria and low income. Not using condoms was associated with women being more likely to agree to have sex after they drink. Programs to increase contraception use among this high risk group may need to address alcohol use and target women who may be economically disadvantaged. Further studies are needed to investigate the contraceptive behaviors among participants and the role that alcohol use and economic constrains may play in women's patterns of contraceptive use and abortion rates.

## Competing interests

The authors declare that they have no competing interests.

## Authors' contributions

NA: main contributor to conception, statistic analyses and writing. WZ: contributed to data analysis and interpretation of the data. AS: contributed to data acquisition, coordination and management, as well as quality control and interpretation of data. RS: contributed to data acquisition, coordination and management, as well as quality control and interpretation of data. AK: contributed to conception and review of the manuscript. TK: contributed to data acquisition, quality control and interpretation and management of data. All authors read and approved the final manuscript.

## References

[B1] Popov AA Family planning in the ussr. Sky-high abortion rates reflect dire lack of choice Entre Nous Cph Den 1990 5 7 12222340

[B2] CDC and ORC Macro Reproductive, maternal and child health in eastern europe and eurasia: A comparative report Atlanta, GA, Population Reference Bureau 2003

[B3] Ipas: Russia Reproductive health and abortion statistics Chapel Hill, NC, International Data for Evaluation of Abortion Services 2007 Population Reference Bureau

[B4] Regushevskaya E Dubikaytis T Laanpere M Nikula M Kuznetsova O Haavio-Mannila E Karro H Hemminki E Risk factors for induced abortions in st petersburg, estonia and finland. Results from surveys among women of reproductive age Eur J Contracept Reprod Health Care 2009 14 176 186 10.1080/13625180902916038 19565415

[B5] Talykova LV Vaktskjold A Serebrjoakova NG Khokhlov TV Strelkovskaja NJ Chashchin VP Nikanov AN Odland JO Bykov V Nieboer E Pregnancy health and outcome in two cities in the kola peninsula, northwestern russia Int J Circumpolar Health 2007 66 168 181 17515256 10.3402/ijch.v66i2.18249

[B6] Parfitt T Russia moves to curb abortion rates Lancet 2003 362 968 10.1016/S0140-6736(03)14404-2 14513847

[B7] Fedorova GV Baniushevich IA [factors impacting abortion prevalence] Probl Sotsialnoi Gig Zdravookhranenniiai Istor Med 2005 19 23 16821309

[B8] Karelova GN A reduction of abortions. Russian federation The hague forum. Integration 1999 29 12322188

[B9] Sherwood-Fabre L Goldberg H Bodrova V The impact of an integrated family planning program in russia Eval Rev 2002 26 190 212 10.1177/0193841X02026002003 11949538

[B10] Perlman F McKee M Trends in family planning in russia, 1994-2003 Perspect Sex Reprod Health 2009 41 40 50 10.1363/4104009 19291128 PMC3071936

[B11] David PH Reichenbach L Savelieva I Vartapetova N Potemkina R Women's reproductive health needs in russia: What can we learn from an intervention to improve post-abortion care? Health Policy Plan 2007 22 83 94 10.1093/heapol/czm003 17299022

[B12] UNAIDS/WHO Aids epidemic update | global report Geneva 2010

[B13] Ministry of Health and Social Development of the Russian Federation Country progress report of the russian federation on the implementation of the declaration of commitment on hiv/aids: Moscow 2008

[B14] Abdala N Kershaw T Krasnoselskikh TV Kozlov AP Contraception use and unplanned pregnancies among injection drug-using women in st petersburg, russia J Fam Plann Reprod Health Care 2011 37 158 164 10.1136/jfprhc-2011-0079 21493618 PMC3636722

[B15] Perlman FJ Drinking in transition: Trends in alcohol consumption in russia 1994-2004 BMC Public Health 2010 10 691 10.1186/1471-2458-10-691 21070625 PMC2997093

[B16] Pomerleau J McKee M Rose R Haerpfer CW Rotman D Tumanov S Drinking in the commonwealth of independent states--evidence from eight countries Addiction 2005 100 1647 1668 10.1111/j.1360-0443.2005.01233.x 16277626

[B17] Stickley A Leinsalu M Andreev E Razvodovsky Y Vagero D McKee M Alcohol poisoning in russia and the countries in the european part of the former soviet union, 1970 2002 Eur J Public Health 2007 17 444 449 17327281 10.1093/eurpub/ckl275

[B18] Hingson R Heeren T Winter MR Wechsler H Early age of first drunkenness as a factor in college students' unplanned and unprotected sex attributable to drinking Pediatrics 2003 111 34 41 10.1542/peds.111.1.34 12509551

[B19] Lavikainen HM Lintonen T Kosunen E Sexual behavior and drinking style among teenagers: A population-based study in finland Health Promot Int 2009 24 108 119 10.1093/heapro/dap007 19304992

[B20] Lysova AV Hines DA Binge drinking and violence against intimate partners in russia Aggress Behav 2008 34 416 427 10.1002/ab.20256 18384157

[B21] Somlai AM Kelly JA McAuliffe TL Ksobiech K Hackl KL Predictors of hiv sexual risk behaviors in a community sample of injection drug-using men and women AIDS Behav 2003 7 383 393 14707535 10.1023/b:aibe.0000004730.62934.ed

[B22] Bradley KA DeBenedetti AF Volk RJ Williams EC Frank D Kivlahan DR Audit-c as a brief screen for alcohol misuse in primary care Alcohol Clin Exp Res 2007 31 1208 1217 10.1111/j.1530-0277.2007.00403.x 17451397

[B23] Frank D DeBenedetti AF Volk RJ Williams EC Kivlahan DR Bradley KA Effectiveness of the audit-c as a screening test for alcohol misuse in three race/ethnic groups J Gen Intern Med 2008 23 781 787 10.1007/s11606-008-0594-0 18421511 PMC2517893

[B24] Jukkala T Makinen IH Kislitsyna O Ferlander S Vagero D Economic strain, social relations, gender, and binge drinking in moscow Soc Sci Med 2008 66 663 674 10.1016/j.socscimed.2007.10.017 18023952

[B25] Tomkins S Saburova L Kiryanov N Andreev E McKee M Shkolnikov V Leon DA Prevalence and socio-economic distribution of hazardous patterns of alcohol drinking: Study of alcohol consumption in men aged 25-54 years in izhevsk, russia Addiction 2007 102 544 553 10.1111/j.1360-0443.2006.01693.x 17362291 PMC1890567

[B26] Aicken CR Nardone A Mercer CH Alcohol misuse, sexual risk behaviour and adverse sexual health outcomes: Evidence from britain's national probability sexual behaviour surveys J Public Health (Oxf) 2011 33 262 271 10.1093/pubmed/fdq056 20705716

[B27] Flanigan BJ Hitch MA Alcohol use, sexual intercourse, and an exploratory study J Alcohol Drug Educ 1986 31 6 40 12268502

[B28] Abdala N White E Toussova OV Krasnoselskikh TV Verevochkin S Kozlov AP Heimer R Comparing sexual risks and patterns of alcohol and drug use between injection drug users (idus) and non-idus who report sexual partnerships with idus in st. Petersburg, russia BMC Public Health 2010 10 676 10.1186/1471-2458-10-676 21054855 PMC2988741

[B29] WHO Alcohol use and sexual risk behaviour: A cross-cultural study in eight countries Department of Mental Health and Substance Abuse-Geneva 2005

[B30] Rossow I Romelsjo A The extent of the 'prevention paradox' in alcohol problems as a function of population drinking patterns Addiction 2006 101 84 90 10.1111/j.1360-0443.2005.01294.x 16393194

[B31] Leon DA Saburova L Tomkins S Andreev E Kiryanov N McKee M Shkolnikov VM Hazardous alcohol drinking and premature mortality in russia: A population based case-control study Lancet 2007 369 2001 2009 10.1016/S0140-6736(07)60941-6 17574092

[B32] Gmel G Bissery A Gammeter R Givel JC Calmes JM Yersin B Daeppen JB Alcohol-attributable injuries in admissions to a swiss emergency room--an analysis of the link between volume of drinking, drinking patterns, and preattendance drinking Alcohol Clin Exp Res 2006 30 501 509 10.1111/j.1530-0277.2006.00054.x 16499491

[B33] Rose AK Grunsell L The subjective, rather than the disinhibiting, effects of alcohol are related to binge drinking Alcohol Clin Exp Res 2008 32 1096 1104 10.1111/j.1530-0277.2008.00672.x 18445111

[B34] Regushevskaya E Dubikaytis T Nikula M Kuznetsova O Hemminki E Contraceptive use and abortion among women of reproductive age in st. Petersburg, russia Perspect Sex Reprod Health 2009 41 51 58 10.1363/4105109 19291129

[B35] Weinhardt LS Carey MP Does alcohol lead to sexual risk behavior? Findings from event-level research Annu Rev Sex Res 2000 11 125 157 11351830 PMC2426779

[B36] Mathias AS Turrentine CG An intimate look at contraception and alcohol consumption NASPA Journal 2003 40 105 123

[B37] Savelieva I Pile JM Sacci I Loganathan R Postabortion family planning operations research study in perm, russia Atlanta, GA, Frontiers in Reproductive Health 2003

